# Structural insights into RAB7A activation: a mammalian perspective on the MON1A–CCZ1 GEF complex

**DOI:** 10.1093/lifemeta/loaf021

**Published:** 2025-06-09

**Authors:** Life Metabolism Editorial Team

Autophagy ensures cellular homeostasis through lysosome-mediated degradation, especially under stress conditions [1]. The maturation and fusion of autophagosomes with lysosomes constitute the final and critical step of the autophagic process, tightly orchestrated by a subset of RAB family small GTPases [2]. Among them, RAB7A functions as a master regulator of late endosomal-lysosomal trafficking, including autophagosome maturation, lysosome positioning, and membrane fusion [3]. Like other RAB GTPases, the activity of RAB7A is dynamically controlled by a GTP/GDP switch mechanism orchestrated by guanine nucleotide exchange factors (GEFs) and GTPase-activating proteins (GAPs) [4].

In fungi, the RAB7 ortholog Ypt7 is activated by the Mon1–Ccz1 (MC1) complex, a heterodimeric GEF that catalyzes the exchange of GDP for GTP [5]. The MC1 complex comprises multiple longin domains (LDs), which contribute to membrane targeting and RAB specificity. The MC1 complex is evolutionarily conserved across eukaryotic species from yeast to *Drosophila* and plants, functioning as a GEF for RAB7 orthologs. While the core architecture and GEF function are conserved, higher eukaryotes exhibit additional regulatory elements, such as RMC1 (regulator of MON1–CCZ1) in mammals, reflecting the increasing complexity of endolysosomal trafficking and signaling networks [6].

Despite significant advances in structural insights through cryo-electron microscopy (cryo-EM) and crystallography of fungal and insect MC1 complexes [6–9], the precise mechanism of RAB7A activation in mammals has remained poorly understood. A recent study has reported a cryo-EM structure of the human MON1A–CCZ1–C18orf8–RAB7A complex, capturing RAB7A in its GDP-bound T22N form [10], however, this conformation does not accurately reflect the nucleotide exchange intermediate and leaves unresolved key catalytic features, such as the dynamics of the nucleotide-binding pocket and the role of metazoan-specific elements.

A recent study published in *Life Metabolism* addresses this gap by presenting a high-quality structural reconstruction of the human HsMC1–RAB7A^N125I^ complex, overcoming a long-standing challenge in protein expression and complex stabilization by optimizing a truncated MON1A construct and co-expressing the components in insect cells. The study for the first time captures the high-resolution cryo-EM structure of the human MON1A–CCZ1 (HsMC1) complex bound to nucleotide-free RAB7A (N125I mutant) at a resolution of 2.85 Å [11], revealing a previously unrecognized interaction between CCZ1 and the phosphate-binding loop ( P-loop) of RAB7A. This unprecedented interaction redefines the new role of P-loop, a conserved structural element found in nucleotide-binding proteins, as a dynamic regulatory hub for GEF-mediated activation, complementing the canonical remodeling of the Switch I region [12]. Structure-guided mutagenesis and *in vitro* GEF assay further confirm the functional importance of this interface and identify the metazoan-conserved residues as essential determinants of GEF activity.

While previous models based on yeast and *Drosophila* have provided some foundational insights into MC1 complexes, the identification of metazoan-specific elements in the HsMC1 complex, such as distinct interface residues and regulatory loops, as well as the presence of auxiliary subunits like RMC1 in mammals which are absent in lower organisms, suggests an evolutionary strategy to fine-tune GEF activity. Future work will be needed to investigate how other cofactors, including RMC1 or membrane lipids, modulate the activity of the HsMC1 complex.

**Figure 1.**
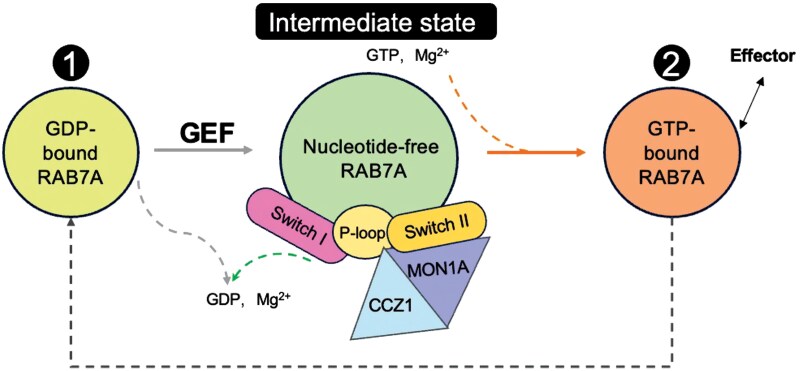
This schematic diagram illustrates the transition cycle of RAB7A between its inactive and active states. In its inactive form, RAB7A is bound to GDP (yellow circle). The MON1A−CCZ1 complex, acting as a GEF, catalyzes the release of GDP upon binding to RAB7A, generating a nucleotide-free intermediate (green circle). During this process, key structural motifs, including the Switch I, the P-loop, and the Switch II, undergo conformational changes. Association with GTP and Mg²⁺ activates RAB7A (orange circle), enabling effector recognition and downstream signaling. Subsequent GTP hydrolysis reverts RAB7A to the GDP-bound state, completing the cycle.

Given the essential role of RAB7A in autophagosome maturation, lysosomal function, and endosomal trafficking, this structural framework could inform the design of targeted therapeutics for diseases marked by autophagy dysfunction, including neurodegenerative disorders and metabolic syndromes. The mechanistic model of GDP displacement, involving conformational rearrangements that dislodge Mg^2+^ and promote GTP loading, could serve as a conceptual scaffold for identifying regulatory checkpoints within autophagic flux (Fig. 1). In summary, the cryo-EM structure of the HsMC1–RAB7A complex elucidates the molecular mechanism of nucleotide exchange within a physiologically relevant intermediate state and provides a robust structural framework for further investigation.
